# Depleted circulatory complement-lysis inhibitor (CLI) in childhood cerebral malaria returns to normal with convalescence

**DOI:** 10.1186/s12936-020-03241-5

**Published:** 2020-04-26

**Authors:** Samuel Eneọjọ Abah, Florence Burté, Steven A. Howell, Ikeoluwa Lagunju, Wuraola A. Shokunbi, Mats Wahlgren, Olugbemiro Sodeinde, Biobele J. Brown, Anthony A. Holder, Delmiro Fernandez-Reyes

**Affiliations:** 1 Francis Crick Institute, 1 Midland Road, London, NW1 1AT UK; 2 Department of Paediatrics, College of Medicine, University of Ibadan, University College Hospital, Ibadan, Nigeria; 3 Childhood Malaria Research Group, College of Medicine, University of Ibadan, University College Hospital, Ibadan, Nigeria; 4 Department of Haematology, College of Medicine, University of Ibadan, University College Hospital, Ibadan, Nigeria; 5 Department of Microbiology, Tumour and Cell Biology, Karolinska Institutet, Stockholm, Sweden; 6 Department of Computer Science, Faculty of Engineering, University College London, Gower Street, London, WC1E 6BT UK

**Keywords:** Pathogenesis, Childhood severe malaria, Cerebral malaria, Biomarkers

## Abstract

**Background:**

Cerebral malaria (CM), is a life-threatening childhood malaria syndrome with high mortality. CM is associated with impaired consciousness and neurological damage. It is not fully understood, as yet, why some children develop CM. Presented here is an observation from longitudinal studies on CM in a paediatric cohort of children from a large, densely-populated and malaria holoendemic, sub-Saharan, West African metropolis.

**Methods:**

Plasma samples were collected from a cohort of children with CM, severe malarial anaemia (SMA), uncomplicated malaria (UM), non-malaria positive healthy community controls (CC), and coma and anemic patients without malaria, as disease controls (DC). Proteomic two-dimensional difference gel electrophoresis (2D-DIGE) and mass spectrometry were used in a discovery cohort to identify plasma proteins that might be discriminatory among these clinical groups. The circulatory levels of identified proteins of interest were quantified by ELISA in a prospective validation cohort.

**Results:**

The proteome analysis revealed differential abundance of circulatory complement-lysis inhibitor (CLI), also known as Clusterin (CLU). CLI circulatory level was low at hospital admission in all children presenting with CM and recovered to normal level during convalescence (p < 0.0001). At acute onset, circulatory level of CLI in the CM group significantly discriminates CM from the UM, SMA, DC and CC groups.

**Conclusions:**

The CLI circulatory level is low in all patients in the CM group at admission, but recovers through convalescence. The level of CLI at acute onset may be a specific discriminatory marker of CM. This work suggests that CLI may play a role in the pathophysiology of CM and may be useful in the diagnosis and follow-up of children presenting with CM.

## Background

Malaria is one of the major infectious disease challenges in sub-Saharan Africa, particularly in Nigeria. The global burden of malaria in 2017 was estimated to be 219 million cases with 435,000 deaths [1]. The majority of the malaria burden occurs in Africa constituting 92% of the total global cases, with sub-Saharan Africa accounting for nearly 80% of the total malaria burden [1]. Nigeria is foremost of the top five countries that account for nearly half of all malaria cases worldwide [1]. The burden of malaria is predominantly in children who are less than 5 years of age due to *Plasmodium falciparum* severe malaria syndromes [2, 3].

It is still unknown why some children develop severe malaria complications, including cerebral malaria (CM) and severe malaria anaemia (SMA), whilst in others the infection may result in mild or uncomplicated malaria (UM). Childhood CM is a poorly-understood life-threatening syndrome where several complex pathophysiological processes such as mechanical obstruction of cerebral microvasculature by infected red blood cells [4, 5], neurological dysfunction due to hypoxia [4] and inflammation, and increased blood–brain barrier permeability [2, 4–7] have been proposed to be at play.

Cerebral malaria is clinically defined by an unarousable coma lasting for at least 1 h with or without generalized convulsions, circulatory asexual stages of *P. falciparum*, normal cerebrospinal fluid, and no other cause of encephalopathy [2, 5]. However, its specific diagnosis is extremely challenging in high-transmission, holoendemic malaria regions where other causes of convulsion, such as meningitis and viral encephalitis, may also occur in the presence of circulatory malaria parasites [8]. In this context, there is an urgent need to identify biomarkers that can increase the specificity to differentiate CM at presentation from other encephalopathy-like syndromes, as well as contributing to dissecting the processes involved in its complex pathogenesis. To address this challenge, the circulatory interactions occurring from the onset of severe malaria through to convalescence were studied by carrying out proteomic analyses of plasma from children living in the urban, densely-populated, all-year-round high-transmission of *P. falciparum* malaria setting of the city of Ibadan in sub-Saharan West Africa.

The report shows that circulatory complement-lysis inhibitor (CLI), also known as Clusterin (CLU), is highly depleted in severe malaria. A very low plasma CLI level is associated with children presenting with CM, which recovers with convalescence in the entire CM cohort. More importantly, the circulatory CLI differentiates the CM group from other clinical manifestations of malaria, as well as from malaria-negative encephalopathy-like syndromes with CM-like pathology, including convulsions and meningitis.

## Methods

### Ethical approval

The Ethics Committee at the Institute for Advanced Medical Research and Training of the College of Medicine, University of Ibadan, Nigeria, reviewed the study and gave ethical approval for the sample collection from the hospital platform, primary care centres and schools in the city of Ibadan, Nigeria. Parents and/or guardians of study participants gave informed written consent in accordance with the World Medical Association’s ethical principles for research involving human subjects.

### Study site

All study participants were recruited from 2008 to 2013 at the University College Hospital (UCH) in the city of Ibadan, Nigeria, a densely populated malaria-hyperendemic city [9]. It has an 8-month rainy season [10], with more than 10 rainy days per month between May and October [2]. There is little rainfall between November and March, a period that includes the harmattan season, characterized by a hot, dry and dusty northeast wind. Malaria transmission and the resultant severe disease occur throughout the year [10]. Out of 16,031 children recruited in Ibadan over a period of 6 years, severe malaria constituted 1806 (11.3%) cases and about three-quarters (75.3%) of all severe malaria cases were reported to be associated with severe anaemia, while cases of CM constituted 19.7% [9]. Although severe malaria syndromes are predominant in children under 5 years-of-age, there is a large and significant burden of severe malaria in children up to 16 years-of-age in Ibadan [2, 11].

### Subjects and case definitions

This study was part of the larger case–control study on severe malaria as reported previously [2, 3, 10–12]. Patient demographics are shown in Tables 1 and 2. Well-defined malaria-positive clinical cases were placed into three groups as UM, SMA and CM in accordance with World Health Organization (WHO) criteria [2, 10, 13, 14]. The UM cases were defined as patients with fever and *P. falciparum* parasitaemia who did not require hospital admission and were recruited as part of a daily malaria parasite screening routine at children outpatient clinics. The SMA group was comprised of conscious children with a packed cell volume (PCV) of less than 16% or a hemoglobin concentration of less than 5 g/dl, with asexual forms of *P. falciparum* detected in blood films and no other evident cause of the anaemia. The CM group consisted of children with an unarousable coma that persisted for more than an hour, with generalized convulsions and the presence of asexual forms of *P. falciparum* and normal cerebrospinal fluid. Coma status was defined by a Blantyre coma score < 2 [2, 10, 15]. Two additional control groups were also recruited: the Disease Control (DC) cohort, consisted of parasite-negative children with anaemia, convulsions, encephalopathy-like syndrome and meningitis; symptoms that closely mimic those of CM/SMA; and the healthy Community Control (CC) cohort comprised of healthy children who were parasite negative.

**Table 1:** Clinical characteristics of the proteomics discovery cohort

Proteomics discovery cohort	Clinical groups [Total N = 120]			
	UM	CM	SMA	CC
Pool N (%)	30 (25%)	30 (25%)	30 (25%)	30 (25%)
Age (months) Median (IQR)	43 (28–81)	48 (36–62)	39 (19–58)	72 (37–96)
Sex: F/M	18/12	15/15	16/14	20/10
PCV (%) Median (IQR)	31 (25–35)	25 (20–31)	12 (11–15)	34 (32–35)
Parasite density^a^ Median (IQR)	45,981 (19,506–61,646)	2252 (667–65,791)	17,216 (1193–30,806)	N/A

^a^ Parasite density not significantly different between malaria groups

*N* number, *IQR* interquartile range, *PCV* pack cell volume, *UM* uncomplicated malaria, *SMA* severe malarial anaemia, *CM* cerebral malaria

**Table 2:** Clinical characteristics of CLI validation cohort

Validation cohort	Clinical groups [Total N = 177 at onset; N = 61 at recovery]				
	UM	CM	SMA	DC	CC
Onset N (%)	38 (16.3%)	35 (24.5%)	30 (16.3%)	39 (11%)	35 (27.3%)
Recovery N (%)	18 (36%)	25 (32%)	18 (32%)	N/A	N/A
Age (months) Median (IQR)	45 (6–145)	49 (15–165)	37 (6–150)	55 (5–150)	50 (10–100)
Sex: F/M	24/14	20/15	14/16	19/20	20/15
PCV (%) At onset Median (IQR)	32 (27–35)	25 (23–30)	13 (11–15)	14 (8–23)	34 (32–35)
PCV (%) At recovery Median (IQR)	35 (29–40)	34 (27–40)	34 (27–37)	35 (28–)	35 (27–40)
Parasite density At onset^a^ Median (IQR)	27,429 (1901–54,680)	5590 (1314–81,923)	32,131 (2487–103,822)	N/A	N/A

^a^Parasite density not significantly different between malaria groups

*N* number, *IQR* interquartile range, *PCV* pack cell volume, *UM* uncomplicated malaria, *SMA* severe malarial anaemia, *CM* cerebral malaria

All children diagnosed with severe malaria syndromes were admitted to the emergency ward and those with severe anaemia were transfused within 2 h of admission and confirmation of malaria diagnosis. However, samples were collected prior to the blood transfusion. All children diagnosed with malaria were treated and followed through convalescence to recovery following Nigerian anti-malarial treatment guidelines, which are based on the WHO guidelines [16]. Uncomplicated malaria was treated with a 3-day oral course of artemisinin-based combination therapy (ACT). On the other hand, severe malaria was treated with intravenous artesunate for at least 24 h and until the patient could tolerate oral therapy, after which treatment was then completed with 3 days of oral ACT. Overall, successful anti-malarial treatment was completed in less than 1 week. In the clinics at UCH Ibadan, an urban academic tertiary-care centre, guidelines are strictly followed and monitored weekly.

### Clinical data and malaria diagnosis

Clinical data were collected using a malaria-focused questionnaire designed by the Childhood Malaria Research Group (CMRG) at UCH Ibadan. Malaria parasites (MPs) were detected and counted by microscopy following Giemsa-staining of thick and thin blood films. The criterion for declaring a participant to be malaria parasite-free was no detectable parasites in 100 high-power (1000×) fields in both thick and thin films [2, 10]. This diagnostic procedure was validated by randomly selecting one in ten thick blood films for independent review by local experienced senior microscopists who were not part of the study research team. Parasite density was computed from the enumeration of malaria parasites that were detected by microscopy. Packed cell volume (PCV) was measured using the microhaematocrit method. Briefly, blood was collected in a capillary tube and then the tube was centrifuged at 12,000*g* for 5 min. The cell volume was calculated as a percentage of the whole tube volume [2].

### Plasma sample collection

Plasma samples were collected at recruitment and following recovery for the malaria-positive patients. The first samples collected on the day of recruitment or hospital admission, prior to drug treatment or blood transfusion, were labeled as onset. Samples following recovery were collected between 28 and 35 days after recruitment and were designated as ‘recovery’. A 2.5 ml blood sample was obtained from each participant in an EDTA blood tube and transferred on ice to the central malaria laboratory at UCH. Plasma was harvested immediately following centrifugation (1000*g*, 10 min) and frozen in aliquots at − 80 °C.

### Two-dimensional difference gel electrophoresis (2D-DIGE)

Plasma samples collected from children aged between 6 and 165 months were pooled based on the clinical groups (CM, SMA, UM and CC, n = 30 for each clinical group). For crude plasma analysis, pools were directly processed by 2D-DIGE. For immunodepleted plasma, pooled samples were passed through Seppro IgY14^®^ columns following the manufacturer’s instructions (Sigma-Aldrich, UK), and the combined flow-through and first wash were retained for analysis by 2D-DIGE.

Typically, 120 µg protein was labelled with CyDye DIGE Fluor Labeling Kit, following the manufacturer’s instructions (GE Healthcare Life Science). The Cy5 and Cy3 dyes were used to label pooled plasma proteins from two different disease groups, whilst Cy2 was used to label proteins from a pool of plasma samples combining CC, UM, SMA and CM groups to allow comparison between gels. For each gel, all three labeled samples were combined and buffered in DIGE Isoelectric focusing (IEF) buffer (8 M urea, 4% [w/v] CHAPS, 0.4% [v/v] carrier ampholyte, 0.0004% [w/v] bromophenol blue and 130 mM DTT) to a total of 200 µL. These mixed samples were applied onto ReadyStrip IPG strips (pH 3–10, 11 cm, Bio-Rad, UK) and actively rehydrated for 13 h 40 min at 50 V followed by IEF with the voltage ramping option (250 V for 15 min, linear; 8000 V for 2 h, linear; 8000 V for 35,000 V/h, rapid), using a PROTEAN IEF cell (Bio-Rad, UK). The IPG strips were then immediately prepared for SDS-PAGE by transfer into equilibration buffer (50 mM Tris–HCl, pH 8.8 containing 6 M urea; 2% [w/v] SDS; 2% [w/v] DTT and 20% [v/v] glycerol) for 15 min followed by alkylation by the addition of 2.5% (w/v) iodoacetamide for 15 min.

Following SDS-PAGE, the gels were digitally imaged prior to staining with CooBlue following the manufacturer’s instructions (Interchim, UK). The 2D-DIGE pattern was analysed using Bio-Rad PDQuest software (Bio-Rad, UK). Protein spots were defined as being differentially abundant between the pooled samples if they were statistically different using a two-tailed unpaired t-test between groups (n = 4), and a difference of greater than 1.5-fold abundance was detected (Table 3). The Coomassie-stained gel was aligned with the 2D-DIGE images for spot selection and excision; the PDQuest (Bio-Rad, UK) software was used to identify and excise spots of interest using EXQuest spot cutter (Bio-Rad, UK). Protein spots were then processed for mass spectrometry.

**Table 3:** Differentially expressed proteins between malaria groups using 2D-DIGE analysis

Spot	Gel analysis^a^	ID	Protein identification^b^	Mean intensity^c^				log_2_ (FC) compared to CC		
				CC	UM	SMA	CM	UM	SMA	CM
*Fibrinogen subunits*										
1	Crude plasma	P02675	Fibrinogen beta chain	326,043	2,395,644	188,428	342,817	2.88	− 0.79	0.07
23	Immunodepleted	P02671	Fibrinogen alpha chain	111,201	202,297	235,418	193,944	0.86	1.08	0.80
24	Immunodepleted	P02671	Fibrinogen alpha chain	95,490	166,776	248,306	184,968	0.80	1.38	0.95
26	Immunodepleted	P02679	Fibrinogen gamma chain	89,777	167,685	193,504	177,522	0.90	1.11	0.98
*Acute phase proteins*										
2	Crude plasma	P01011	Alpha-1 antichymotrypsin	8437	38,660	53,706	46,532	2.20	2.67	2.46
3	Crude plasma	P01011	Alpha-1 antichymotrypsin	25,637	47,774	54,947	42,469	0.90	1.10	0.73
8	Crude plasma	P02764	Alpha-1-acid glycoprotein 1	194,326	326,673	691,483	742,110	0.75	1.83	1.93
9	Crude plasma	P02764 P19652	Alpha-1-acid glycoprotein 1/2	48,800	193,249	327,999	515,003	1.99	2.75	3.40
21	Immunodepleted	P01011	Alpha-1 antichymotrypsin	563,120	1,142,798	2,002,447	1,967,430	1.02	1.83	1.80
31	Immunodepleted	P02750	Leucine-rich alpha-2 glycoprotein	79,647	217,512	231,940	200,776	1.45	1.54	1.33
*Cholesterol transport*										
29	Immunodepleted	P06727	Apolipoprotein AIV	107,491	54,999	395,132	249,844	− 0.97	1.88	1.22
30	Immunodepleted	P06727	Apolipoprotein AIV	31,611	19,929	45,626	115,303	− 0.67	0.53	1.87
*Heme-induced oxidative stress*										
4	Crude plasma	P00738	Haptoglobin beta chain	121,132	102,981	21,374	88,732	− 0.23	− 2.50	− 0.45
5	Crude plasma	P00738	Haptoglobin beta chain	122,286	176,632	0	114,007	0.53		− 0.10
6	Crude plasma	P00738	Haptoglobin beta chain	136,742	192,620	35,403	147,950	0.49	− 1.95	0.11
7	Crude plasma	P00738	Haptoglobin beta chain	89,871	158,852	29,216	126,023	0.82	− 1.62	0.49
10	Crude plasma	P00738	Haptoglobin alpha-2-chain	51,148	78,021	0	25,545	0.61		− 1.00
11	Crude plasma	P00738	Haptoglobin alpha-2-chain	20,545	78,021	0	2890	1.93		− 2.83
15	Crude plasma	P68871	Haemoglobin subunit-beta	47,555	26,914	168,212	15,023	− 0.82	1.82	− 1.66
16	Crude plasma	P69905	Haemoglobin subunit-alpha	32,203	42,761	278,353	102,665	0.41	3.11	1.67
17	Immunodepleted	P02790	Hemopexin	1,712,652	1,368,897	327,700	812,479	− 0.32	− 2.39	− 1.08
18	Immunodepleted	P02790	Hemopexin	57,181	41,615	26,793	24,527	− 0.46	− 1.09	− 1.22
19	Immunodepleted	P02790	Hemopexin	601,982	411,670	171,318	345,268	− 0.55	− 1.81	− 0.80
20	Immunodepleted	P02790	Hemopexin	1,788,743	1,276,004	698,022	754,038	− 0.49	− 1.36	− 1.25
*Complement-induced oxidative stress*										
32	Immunodepleted	P10909	Clusterin	47,055	31,176	18,480	30,678	− 0.59	− 1.35	− 0.62

*ID* Protein Accession Number

**Fig. 1:**
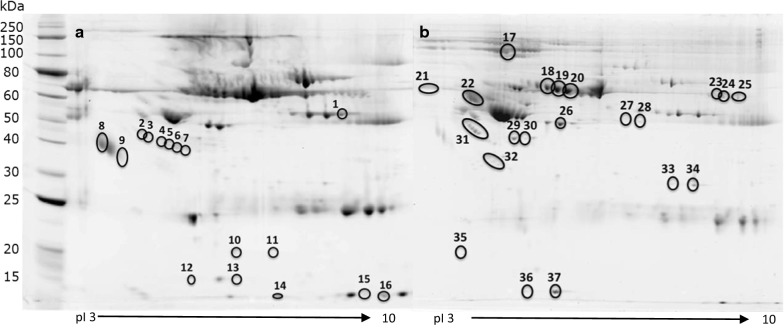
2D-gel electropherograms showing differentially abundant protein spots. Representative images of 2D-gel electropherograms from crude plasma samples (**a**) and immunodepleted plasma samples (**b**). Numbered circled spots represent protein spots showing a differential intensity between groups of over a 1.5-fold-change. Identities and intensity fold-change are depicted in Table 3. *kDa* molecular weight ladder in kDa, *pI* isoelectric point

^a^Spot detected as > 1.5-fold significant change compared to other groups identified and analysed from either crude plasma sample or an immunodepleted plasma sample. Representative images and positions of numbered spots are shown in Fig. 1

^b^Spot identifications are detailed in Additional file 1: Fig. S1

^c^Mean intensity as estimated by Bio-Rad PDQuest software. (n = 2 for crude plasma, n = 4 for immunodepleted)

^d^Log_2_ (fold change) mean intensity of protein in each disease case compared to CC

### Protein identification by mass spectrometry

Excised gel spots containing proteins for analysis (Additional file 1: Fig. S1) were washed in 200 mM ammonium bicarbonate (NH_4_HCO_3_), 50% acetonitrile (ACN) for 30 min at 37 °C until all the stain had been removed. Spots were then incubated in 20 mM DTT (prepared in 200 mM NH_4_HCO_3_, 50% ACN) for 1 h at 37 °C and washed in the same solution without DTT, follow by an incubation in 5 mM iodoacetamide (prepared in 200 mM NH_4_HCO_3_, 50% ACN) for 20 min at 37 °C. Gel pieces were dehydrated in 100% ACN for 15 min and the supernatant removed. In-gel digestion was performed by adding 2 µg/ml Trypsin Gold (Promega) at 32 °C for 16 h. Soluble tryptic peptides were analysed by LC–MS/MS using an OrbiTrap LC–MS (Thermo-Scientific). The peptide and fragment ion profiles were extracted and compared to the Mascot database for protein identification.

### Enzyme-linked immunosorbent assay of plasma CLI level

Complement Lysis Inhibitor (CLI) levels in plasma were measured using a human CLI ELISA kit (sold as CLU kit, Ray Biotech. Inc.) and the manufacturer’s procedure. Briefly, the plasma samples stored at − 80 °C prior to the assay were thawed on ice and diluted serially in 1× assay diluent to a final dilution of 1/50,000. CLI standard and diluted samples were added to 96-well plates coated with anti-CLI antibodies and incubated at 4 °C overnight. Unbound proteins were removed by washing and then biotinylated antibody (1×) was added to the plate and incubated for 1 h at room temperature. After another washing step, HRP-streptavidin solution was added for 45 min followed by subsequent washing steps to remove any unbound proteins. The amount of bound HRP was assayed by adding 3,3,5,5, tetramethylbenzidine (TMB) solution for 15 to 20 min, and the reaction was stopped by addition of 50 µl 1 M H_2_SO_4_ stop solution. The absorbance at 450 nm was measured in a FLUorStar Omega (BMG-Labtech) ELISA plate reader. Computer software (MasterPlex ReaderFit v2.0, MiraiBio Group, Hitachi Solutions America) capable of generating four and five parameters logistic (4/5-PL) curve-fit was used to generate standard curves and the absorbance readings for each sample were used to calculate the CLI concentration from the standard curve.

### Multiplex assay for the measurement of cytokines in plasma samples

Plasma cytokines levels were measured to ascertain whether the observed levels of CLI correlated with and/or were regulated by pro-/anti-inflammatory cytokines. The cytokines levels were measured simultaneously using the Bio-Rad seventeen human cytokine Bio-plex bead immunoassay kit, the Bio-Plex 200 System and the manufacturer’s instructions. The amounts of the individual analytes were assayed for each sample using the Bio-Plex Manager 6.0 software (Bio-Rad Laboratories).

### Statistical analysis

**Fig. 2:**
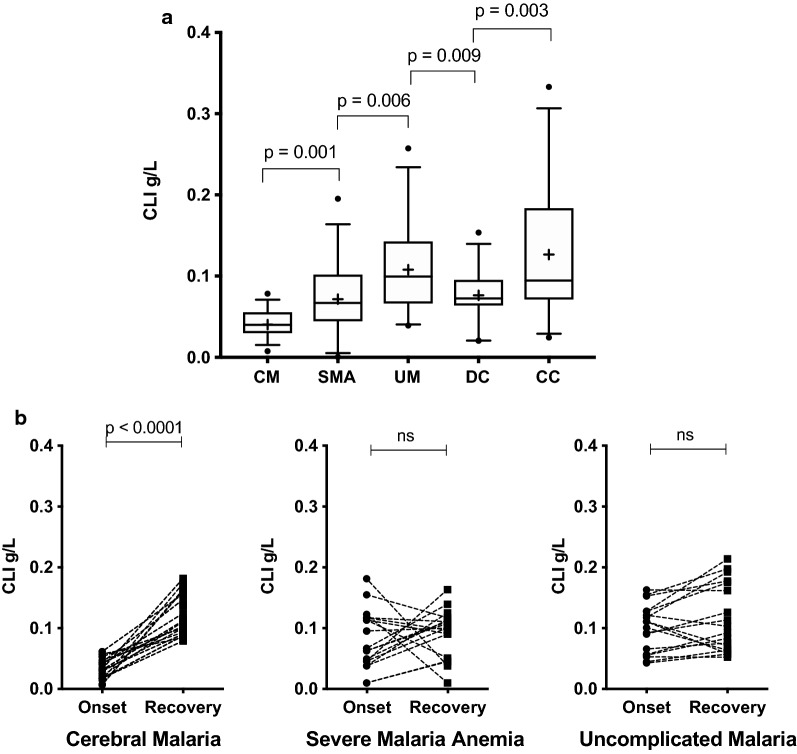
Plasma CLI concentration in clinical malaria syndromes both at acute onset and at recovery. **a** Plasma CLI protein level in clinical malaria syndromes at acute onset CM (0.04 ± 0.01); SMA (0.07 ± 0.04); UM (0.11 ± 0.05); DC (0.07 ± 0.03); CC (0.13 ± 0.08). **b** Two time-point graph showing levels of CLI at onset and recovery (paired samples) in the CM, SMA and UM groups, respectively. *CLI* complement-lysis inhibitor, *CM* = cerebral malaria, *SMA* severe malaria anaemia, *UM* uncomplicated malaria, *DC* disease controls, *CC* community controls; (mean ± standard deviation) g/L

**Fig. 3:**
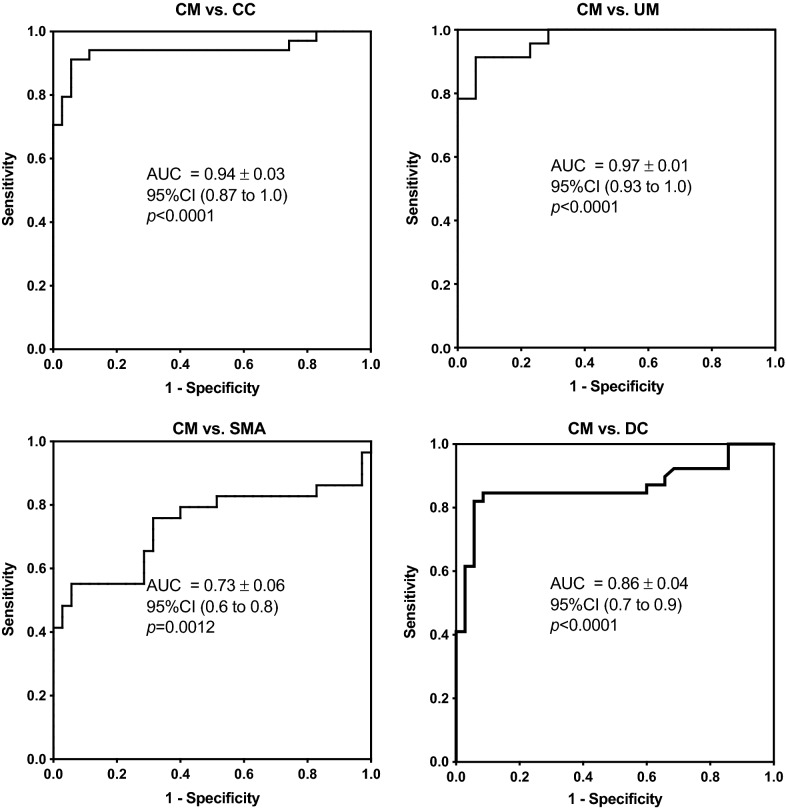
CLI discriminating potential between each malaria syndrome at acute onset or control group using ROC curve analysis. *CLI* complement-lysis inhibitor, *CM* cerebral malaria, *SMA* severe malaria anaemia, *UM* uncomplicated malaria, *DC* disease controls, *CC* community controls, *AUC* area under the curve

For the proteomic analysis, a log_2_-fold change was used to compute differences in protein levels in the disease groups relative to the CC group (Table 3). The CLI measurement study was designed to detect at least a 20% difference in protein level at recruitment and recovery with 80% power and a conventional level of alpha, 0.05. This design was used to reduce the likelihood of making type 1 and 2 errors or detecting an effect in the absence of any, and vice versa. Prism software was used for the statistical analysis. An unpaired t-test (Mann–Whitney test) was used to make comparisons between data from different clinical groups (Fig. 2a) while a paired non-parametric t-test was used to make comparison between data in the same group; for example, data at both recruitment and recovery (Fig. 2b). Non-parametric Spearman correlation analysis was used to correlate CLI levels with those of cytokines (Table 4). In order to evaluate the diagnostic potential of CLI in discriminating CM from SMA, UM, DC and CC groups at admission, the likelihood ratio was computed by plotting sensitivity against 1-specificity in a receiver operating characteristic (ROC) curve analysis and the area under the curve (AUC), the 95% Confidence Interval (CI) and p-values were used to interpret the data (Fig. 3).

**Table 4:** Correlation analysis between plasma CLI and inflammatory cytokines levels

CLI	CM			SMA			UM		
	IL-10	IL-6	IL-8	IL-10	IL-6	IL-8	IL-10	IL-6	IL-8
Onset	r = − 0.533 p = 0.040	r = − 0.622 p = 0.010	r = − 0.499 p = 0.036	r = 0.109 p = ns	r = 0.063 p = ns	r = 0.255 P = ns	r = − 0.657 p = ns	r = 0.029 p = ns	r = 0.1429 p = ns
Recovery	r = 0.043 p = 0.617	r = 0.350 p = ns	r = 0.383 p = ns	r = − 0.559 p = 0.047	r = − 0.784 p = 0.004	r = 0.0608 p = ns	r = 0.714 p = ns	r = 0.371 p = ns	r = 0.314 p = ns

*IL* interleukins, *CLI* complement lysis inhibitor; *UM* uncomplicated malaria, *SMA* severe malarial anaemia, *CM* cerebral malaria, *r* non-parametric spearman correlation coefficient (statistically significant if p < 0.05 according to the non-parametric spearman r-test), *ns* non-significant

## Results

### Study cohort characteristics

A total of 120 plasma samples in the Discovery Cohort (Table 1) were collected at acute onset. The Validation Cohort (Table 2) had plasma samples from a total of 177 patients, which were collected at two sampling time points: at acute onset (at admission) and at recovery (at discharge from hospital). The number of plasma samples collected on recovery in the UM, CM and SMA groups was a little lower than at onset as some patients were missed in the study (Table 2). Malaria parasites were not detected in the DC and CC groups (Tables 1 and 2). Female and male patients were represented in all the clinical groups (Tables 1 and 2). The lowest median age and PCV measurements were in the SMA group but they were not significantly different to those of the UM, CM and control groups (CC and DC).

### Plasma proteomic analysis detects differences in CLI abundance in discovery cohort

The proteomic analysis was designed to detect overall changes in protein levels between malaria disease groups (CM, SMA, UM) and CC. To achieve this, 30 patient samples were pooled together for each disease group (Table 1).

Proteomic profiling of crude pooled plasma samples (Fig. 1a) detected a significant differential abundance of 16 protein spots (Fig. 1a, spots 1 to 16). This approach was extended further by depleting the most abundant proteins prior to gel analysis, allowing the detection of a further 20 differentially abundant proteins (Fig. 1b, spots 17–37). Spot excision followed by tryptic digestion and LC–MS/MS peptide analysis identified 58 proteins (Additional file 1). The proteins differentially abundant between disease groups and the CC group are shown in Table 3. Proteins of potential interest included fibrinogen chains, proteins from the acute phase (α-1-antichymotrypsin, leucine-rich α-2-glycoprotein, α-1-acid glycoprotein), cholesterol transport proteins (Apolipoprotein AIV), haem-induced oxidative stress (haptoglobin chains, haemoglobin chains and hemopexin) and one protein from the complement-oxidative stress pathway, the complement lysis inhibitor (CLI), which was highly depleted in all groups, and which is the focus in this study (Table 3, Additional file 1: Fig. S1).

### Circulatory CLI level in the CM group is low at hospital admission in the validation cohort

CLI changes observed in the discovery cohort were re-examined in a separate validation cohort of individual (non-pooled) samples at disease onset, using an ELISA-based assay (Fig. 2a) with observed CLI (mean ± standard deviation) in g/L as follows: CM (0.04 ± 0.01); SMA (0.07 ± 0.04); UM (0.11 ± 0.05); DC (0.07 ± 0.03); CC (0.13 ± 0.08). The plasma level of CLI was significantly lower in the CM group compared to levels in the SMA, UM, DC and CC groups (Fig. 2a). CLI levels at acute onset in the SMA group were significantly lower than in the UM group (Fig. 2a). Levels in the DC group were significantly lower than those in both CC and UM groups (Fig. 2a). ROC analysis showed that plasma CLI levels in the CM group at acute onset discriminate CM from other malarial syndromes at admission, particularly the UM (AUC = 0.97, 95% CI 0.9335–1.007, p < 0.0001) and the control groups (AUC = 0.94, 95% CI 0.8764–1.006, p < 0.0001 for CC and AUC = 0.86, 95% CI 0.7736–0. 9561, p < 0.0001 for DC, Fig. 3). The SMA and CM groups showed the lowest degree of discrimination (AUC = 0.74, 95% CI 0.6018–8721, p = 0.0012, Fig. 3).

### The low plasma CLI level at hospital admission in the CM group returns to normal with convalescence

The low levels of CLI at acute onset observed in the CM group had returned to normal levels in all the patients following convalescence (Fig. 2b) within 28–35 days from the day of admission. The CLI levels in g/L are shown in (median; IQR; range). The CLI levels in the CM group at recovery (0.11; 0.057; 0.10) were significantly (p < 0.0001) different from the levels in this group at acute onset (0.033; 0.026; 0.055), a pattern not observed in the SMA and UM groups (Fig. 2b). In the UM group, CLI levels at onset (0.11; 0.074; 0.12) were not significantly different to those at recovery (0.098; 0.116; 0.16). Similarly, in the SMA group, CLI levels at onset (0.067; 0.075; 0.17) were not significantly different to those at recovery (0.1; 0.06; 0.15). The SMA and the UM groups did not show a uniform increase in CLI levels from onset to recovery in all patients, unlike the CM group (Fig. 2b). About 16% of the patients in both the UM and the SMA groups had a level of CLI at onset that decreased with recovery (Fig. 2b). A majority of the UM group patients showed a non-significant increase with convalescence (Fig. 2b).

### Plasma CLI level correlates negatively with pro- and anti-inflammatory cytokines in the CM group at hospital admission

At hospital admission (designated as onset), the CM group showed a significant negative correlation in the level of CLI with the pro-inflammatory cytokines IL-6 and IL-8, and the anti-inflammatory cytokine IL-10 (Table 4). At recovery, no significant correlation in levels was observed for CLI and any measured cytokines for the CM group (Table 4). In contrast, for the SMA group at recovery, the CLI levels showed a significant negative correlation with both IL-6 and IL-10 levels (Table 4).

## Discussion

In the context of a large urban, densely-populated, high-transmission, malaria holoendemic setting, the specific diagnosis of CM is extremely challenging, because of the occurrence of other confounding causes of coma and convulsions, such as meningitis and viral encephalitis in the presence of circulatory malaria parasites. To provide solutions to this challenge, well-defined childhood malaria cohorts from the city of Ibadan, Nigeria were studied using well-established plasma proteomic methods.

2D-gel electrophoresis analysis allowed the discrimination of 13 polypeptides corresponding to 9 unique proteins. The pathways involving these proteins are increased coagulation products (fibrinogen subunits), increased acute phase reaction (α-1-antichymotrypsin, α-1-acid-glycoprotein and leucine-rich-α-2 glycoprotein), dysregulation of cholesterol transport (Apolipoprotein IV) and heme-induced oxidative stress (haptoglobin, haemoglobin and hemopexin), all previously established as hallmarks of malaria syndromes [2, 11, 17]. In particular, haptoglobin has been studied further in the present Nigerian cohort [2], showing overall depleted levels in severe malaria groups and the data supporting the idea that low haptoglobinaemia is a risk factor in SMA but not CM. Of particular interest for further analysis here was the identification of the circulatory complement-lysis inhibitor (CLI), also known as Clusterin.

This study reveals that level of CLI is depleted in childhood severe malaria, a finding that is consistent with a previous report of low CLI levels in malaria [18]. The dynamics of CLI levels in each well-defined disease group, and their progression with convalescence is presented to further strengthen the association. This study also shows that CLI level clearly discriminates the malaria and malaria-negative control groups.

Analysis of the discovery cohort used a proteomic approach with pools of plasma to identify proteins with significant differences in abundance. Whilst the approach successfully identified CLI as a protein of interest, it does not allow a detailed analysis of the protein in individual plasma samples or the resolution provided by a specific ELISA-based assay to quantify the protein. Therefore, to analyse the validation cohort, a specific antibody-based assay was used to provide greater specificity and sensitivity and to show that a very low plasma CLI level is uniquely associated with children presenting with CM. The results also show that circulatory CLI levels at acute onset differentiate the CM patient group from patients with other clinical manifestations of malaria as well as malaria-negative encephalopathy-like syndromes with CM-like pathology including convulsions and meningitis with concurrent anaemia. Furthermore, the CLI level in the entire CM patient cohort returned to normal upon recovery, an observation that strengthens the idea of a possible mechanistic role of this multi-faceted protein in the pathogenesis of CM. Moreover, measuring the level of CLI may provide a novel tool to monitor CM disease progression towards convalescence.

Complement-lysis inhibitor is highly conserved between species with 70–80% sequence homology. It binds to IgG, lipids, heparin, bacteria, complement, beta amyloid and leptin [19, 20]. CLI is ubiquitous in most mammalian tissues and it is found in most body fluids [21–25]. It is a multifunctional protein notably important in certain biological functions, such as neuronal protection and cytoprotection, as a secreted chaperone, in cell clustering and aggregation, and as a regulator of inflammatory and complement pathways [26]. These functions are likely to be relevant in the pathophysiology of CM. For example, CLI may act either as a surface bound form or in a diffusible form to protect membranes [27]. Plasma CLI has been reported to accumulate at fluid-tissue boundaries where it has a cytoprotective role [27]. Hence, a plausible role for CLI in CM could be at the blood–brain barrier in regions with membrane destruction due to the cytoadhesion of parasitized red blood cells. There is also evidence of a cytoprotective role of CLI against ischemic injury and hypoxia stress [28]. This protective role of CLI may act through the activation of the Akt signaling pathway and the scavenging of low density lipoprotein (LDL) to prevent oxidative stress [28, 29]. Plasma CLI is known to function as a complement inhibitor by binding complement complexes [30–33]. Complement deposition on erythrocytes is thought to contribute to acute clearance of infected and uninfected erythrocytes in severe malaria [34], further supporting the relevance of CLI plasma levels in CM.

Complement-lysis inhibitor expression is induced by TGF-β and TNF [22, 35]. While no correlation was found between CLI and the level of these cytokines in this study (data not shown), an inverse correlation was observed between CLI and IL-6, IL-8 and IL-10 at onset but not following convalescence in the CM group. This inverse correlation at onset supports a possible association between low levels of CLI and inflammatory events in CM pathophysiology. Mice deficient in CLI are reported to develop severe inflammation [36], providing further evidence of its protective role in inflammation.

Complement-lysis inhibitor depletion may indicate an enhanced demand for the protein or an accelerated scavenging associated with CM pathology. In addition to the potential value of using depleted CLI as a specific CM biomarker at onset of disease and its reconstitution on convalescence, the study suggests that it will be of interest to evaluate further its significance in CM pathophysiology, for which further studies are needed.

## Conclusions

In densely-populated urban cities of sub-Saharan West Africa, with high-transmission all-year-round malaria, childhood populations are under a large burden of severe malaria. In this context, where other causes of coma such as meningitis and viral encephalitis may also occur in the presence of circulatory malaria parasites, there is an urgent need for specific cerebral malaria onset and recovery biomarkers. This study shows that circulatory levels of complement-lysis inhibitor (CLI) can play a role as a specific discriminatory biomarker of CM both at admission as well as during convalescence. These findings also suggest that CLI may play a role in the pathophysiology of CM. Further studies are needed to fully evaluate CLI potential across other regions with varying degrees of malaria disease burden.

## Supplementary information

**Additional file 1.** Supplementary material.

## Data Availability

The datasets during and/or analysed during the current study available from the corresponding author on reasonable request.

## Abbreviations

CLI: Circulatory complement-lysis inhibitor
CM: Cerebral malaria
UM: Uncomplicated malaria
SMA: Severe malaria anaemia
CC: Community control
DC: Disease control
