# Supplementary material for: Depleted circulatory complement-lysis inhibitor (CLI) in childhood cerebral malaria returns to normal with convalescence
Source: Malar J. 2020 Apr 26;19:167. doi: 10.1186/s12936-020-03241-5 (PMC7184698; doi:10.1186/s12936-020-03241-5)
Supplement: Supplementary file 1 — Additional file 1. Supplementary material. [file 12936_2020_3241_MOESM1_ESM.docx]

**Additional Table S1**.

| **Spot** | **Accession** | **Description** | **Score** | **Coverage** | **No. Peptides** | **MW [kDa]** | **calc. pI** |
| --- | --- | --- | --- | --- | --- | --- | --- |
| **ID1** | P02768 | Serum albumin [ALBU] | 6279 | 89.00 | 83 | 69.3 | 6.3 |
| **ID2** | Q14624 | Inter-alpha-trypsin inhibitor heavy chain H4 [ITIH4] | 257 | 14.62 | 14 | 103.3 | 7.0 |
| **ID3** | P02787 | Serotransferrin [TRFE] | 2272 | 63 | 58 | 77.0 | 7.1 |
| **ID4** | P0C0L4 | Complement C4-A beta-chain [CO4A] | 622 | 6.94 | 13 | 72.0 | 8.7 |
| **ID5** | P02768 | Serum albumin [ALBU] | 5752 | 87 | 74 | 69.3 | 6.3 |
| **ID6** | P01042 | Kininogen-1 [KNG1] | 369 | 18.01 | 13 | 71.9 | 6.8 |
|  | P04004 | Vitronectin [VTNC] | 236 | 20.92 | 10 | 54.3 | 5.8 |
| **ID7** | P02765 | Alpha-2-HS-glycoprotein [FETUA] | 655 | 20.16 | 8 | 39.3 | 5.7 |
| **ID8** | P01009 | Alpha-1-antitrypsin [A1AT] | 1004 | 59.09 | 23 | 46.7 | 5.6 |
| **ID9** | P02675 | Fibrinogen beta chain [FIBB] | 4060 | 58.04 | 29 | 55.9 | 8.3 |
| **ID10** | P02675 | Fibrinogen beta chain [FIBB] | 3412 | 53.97 | 24 | 55.9 | 8.3 |
| **ID11** | P01860 | Ig gamma-3 chain C region [IGHG3] | 232 | 0.2732 | 12 | 41.3 | 7.9 |
|  | P01857 | Ig gamma-1 chain C region [IGHG1] | 335 | 0.3909 | 13 | 36.1 | 8.2 |
|  | P01859 | Ig gamma-2 chain C region [IGHG2] | 220 | 0.2301 | 9 | 35.9 | 7.6 |
| **ID12** | P02679 | Fibrinogen gamma chain [FIBG] | 3290 | 0.6954 | 38 | 51.5 | 5.6 |
| **ID13** | P02750 | Leucine-rich alpha-2-glycoprotein [A2GL] | 122 | 12.97 | 5 | 38.2 | 6.9 |
|  | P01011 | Alpha-1-antichymotrypsin [AACT] | 89 | 9.69 | 4 | 47.6 | 5.5 |
| **ID14** | P06727 | Apolipoprotein A-IV [APOA4] | 2498 | 63.64 | 35 | 45.4 | 5.4 |
| **ID15** | O43866 | CD5 antigen-like [CD5L] | 499 | 27.38 | 10 | 38.1 | 5.5 |
| **ID16** | P00738 | Haptoglobin beta-chain [HPT] | 378 | 21.43 | 8 | 45.2 | 6.6 |
| **ID17** | P00738 | Haptoglobin beta-chain [HPT] | 252 | 11.82 | 5 | 45.2 | 6.6 |
| **ID18** | P00738 | Haptoglobin beta-chain [HPT] | 1214 | 35.22 | 16 | 45.2 | 6.6 |
| **ID19** | P00738 | Haptoglobin beta-chain [HPT] | 712 | 31.28 | 14 | 45.2 | 6.6 |
| **ID20** | P25311 | Zinc-alpha-2-glycoprotein [ZA2G] | 254 | 23.73 | 6 | 33.9 | 5.9 |
| **ID21** | P02763 | Alpha-1-acid glycoprotein 1 [A1AG1] | 822 | 33.83 | 8 | 23.5 | 5.0 |
| **ID22** | P02763 | Alpha-1-acid glycoprotein 1 [A1AG1] | 231 | 45.27 | 12 | 23.5 | 5.0 |
| **ID23** | P02763 | Alpha-1-acid glycoprotein 1 [A1AG1] | 822 | 33.83 | 8 | 23.5 | 5.0 |
| **ID24** | P0C0L4 | Complement C4-A gamma-chain [CO4A] | 498 | 5.05 | 11 | 33.0 | 6.4 |
| **ID25** | P02647 | Apolipoprotein A-I [APOA1] | 460 | 40.45 | 11 | 30.8 | 5.8 |
| **ID26** | P02647 | Apolipoprotein A-I [APOA1] | 341 | 56.55 | 22 | 30.8 | 5.8 |
| **ID27** | P02647 | Apolipoprotein A-I [APOA1] | 425 | 67.42 | 25 | 30.8 | 5.8 |
| **ID28** | P01834 | Ig kappa chain C region [IGKC] | 486 | 68.87 | 5 | 11.6 | 5.9 |
|  | P01597 | Ig kappa chain V-I region DEE [KV105] | 107 | 22.22 | 3 | 11.7 | 9.4 |
|  | P01623 | Ig kappa chain V-III region WOL [KV305] | 226 | 33.03 | 4 | 11.7 | 8.9 |
|  | P01842 | Ig lambda chain C regions [LAC] | 133 | 30.48 | 2 | 11.2 | 7.2 |
| **ID29** | P01598 | Ig kappa chain V-I region EU [KV106] | 100 | 26.85 | 2 | 11.8 | 8.4 |
|  | P01834 | Ig kappa chain C region [IGKC] | 489 | 68.87 | 5 | 11.6 | 5.9 |
|  | P06310 | Ig kappa chain V-II region RPMI 6410 [KV206] | 131 | 18.05 | 3 | 14.7 | 9.3 |
|  | P01593 | Ig kappa chain V-I region AG [KV101] | 86 | 27.78 | 2 | 12.0 | 6.0 |
|  | P01597 | Ig kappa chain V-I region DEE [KV105] | 115 | 22.22 | 4 | 11.7 | 9.4 |
|  | P01623 | Ig kappa chain V-III region WOL [KV305] | 212 | 27.52 | 3 | 11.7 | 8.9 |
|  | P01842 | Ig lambda chain C regions [LAC] | 121 | 30.48 | 3 | 11.2 | 7.2 |
|  | P01625 | Ig kappa chain V-IV region Len [KV402] | 48 | 23.68 | 2 | 12.6 | 7.9 |
| **ID30** | P00738 | Haptoglobin alpha-chain [HPT] | 456 | 11.58 | 8 | 16.0 | 5.6 |
| **ID31** | P00738 | Haptoglobin alpha-chain [HPT] | 456 | 11.58 | 8 | 16.0 | 5.6 |
| **ID32** | P02766 | Transthyretin [TTHY] | 284 | 18.37 | 4 | 15.9 | 5.8 |
| **ID33** | P35542 | Serum amyloid A-4 protein [SAA4] | 76 | 15.38 | 2 | 14.8 | 9.2 |
| **ID34** | P68871 | Hemoglobin subunit beta [HBB] | 290 | 55.78 | 7 | 16.0 | 7.3 |
| **ID35** | P01935 | Hemoglobin subunit alpha [HBA] | 345 | 39.72 | 5 | 15.2 | 8.7 |
| **ID36** | P69905 | Hemoglobin subunit alpha [HBA] | 355.47 | 71.83 | 10 | 15.2 | 8.7 |
| **ID37** | P00751 | Complement factor B[CFAB] | 843.09 | 21.99 | 20 | 85.5 | 7.06 |
| **ID38** | P02787 | Serotransferrin [TRFE] | 2272 | 63.18 | 58 | 77 | 7.12 |
| **ID39** | P02790 | Hemopexin OS=Homo sapiens [HEMO] | 482.72 | 38.74 | 24 | 51.6 | 7.02 |
| **ID40** | P02768 | Serum albumin [ALBU] | 5752.43 | 87.19 | 74 | 69.3 | 6.28 |
| **ID41** | P02671 | Fibrinogen alpha chain [FIBA] | 876.52 | 32.68 | 31 | 94.9 | 6.01 |
| **ID42** | P00761 | Alpha-1-antichymotrypsin [AACT] | 505.8 | 32.15 | 14 | 47.6 | 5.52 |
| **ID43** | P01009 | Alpha-1-antitrypsin [A1AT] | 5000.53 | 71.53 | 47 | 46.7 | 5.59 |
| **ID44** | P02675 | Fibrinogen beta chain [FIBB] | 857.68 | 57.03 | 32 | 55.9 | 8.27 |
| **ID45** | P02675 | Fibrinogen beta chain [FIBB] | 2906.97 | 54.99 | 33 | 55.9 | 8.27 |
| **ID46** | P02679 | Fibrinogen gamma chain [FIBG] | 1360.61 | 55.63 | 32 | 51.5 | 5.62 |
| **ID47** | P01860 | Ig gamma-3 chain C region [IGHG3] | 231.6 | 27.32 | 12 | 41.3 | 7.9 |
|  | P01857 | Ig gamma-1 chain C region [IGHG1] | 334.79 | 39.09 | 13 | 36.1 | 8.19 |
|  | P01859 | Ig gamma-2 chain C region [IGHG2] | 219.93 | 23.01 | 9 | 35.9 | 7.59 |
| **ID48** | P01019 | Angiotensinogen [ANGT] | 144.4 | 6.39 | 3 | 53.1 | 6.32 |
| **ID49** | P02765 | Alpha-2-HS-glycoprotein [FETUA] | 324.65 | 15.26 | 8 | 39.3 | 5.72 |
| **ID50** | P02750 | Leucine-rich alpha-2-glycoprotein [A2GL] | 415.25 | 18.16 | 8 | 38.2 | 6.95 |
| **ID51** | P06727 | Apolipoprotein A-IV [APOA4] | 976.5 | 35.86 | 21 | 45.4 | 5.38 |
| **ID52** | P02763 | Alpha-1-acid glycoprotein 1 [A1AG1] | 90.79 | 8.96 | 2 | 23.5 | 5.02 |
| **ID53** | P19652 | Alpha-1-acid glycoprotein 2 [A1AG2] | 201.16 | 11.94 | 5 | 23.6 | 5.11 |
| **ID54** | P10909 | Clusterin [CLUS] | 209.83 | 10.91 | 5 | 52.5 | 6.27 |
| **ID55** | P02741 | C-reactive protein [CRP] | 129.77 | 12.05 | 3 | 25 | 5.63 |
| **ID56** | P01834 | Ig kappa chain C region [IGKC] | 485.52 | 68.87 | 5 | 11.6 | 5.87 |
|  | P01597 | Ig kappa chain V-I region DEE [KV105] | 107.03 | 22.22 | 3 | 11.7 | 9.36 |
|  | P01623 | Ig kappa chain V-III region WOL [KV305] | 226.26 | 33.03 | 4 | 11.7 | 8.91 |
|  | P01625 | Ig kappa chain V-IV region Len [KV402] | 137.3 | 34.21 | 3 | 12.6 | 7.93 |
|  | P01598 | Ig kappa chain V-I region EU [KV106] | 100.17 | 26.85 | 2 | 11.8 | 8.44 |
| **ID57** | P01834 | Ig kappa chain C region [IGKC] | 489.37 | 68.87 | 5 | 11.6 | 5.87 |
|  | P06310 | Ig kappa chain V-II region RPMI 6410 [KV206] | 130.74 | 18.05 | 3 | 14.7 | 9.25 |
|  | P01593 | Ig kappa chain V-I region AG [KV101] | 86.33 | 27.78 | 2 | 12 | 5.99 |
|  | P01597 | Ig kappa chain V-I region DEE [KV105] | 115.42 | 22.22 | 4 | 11.7 | 9.36 |
|  | P01623 | Ig kappa chain V-III region WOL [KV305] | 211.74 | 27.52 | 3 | 11.7 | 8.91 |
|  | P01842 | Ig lambda chain C regions [LAC] | 121.3 | 30.48 | 3 | 11.2 | 7.24 |
| **ID58** | P02766 | Transthyretin [TTHY] | 133.78 | 9.52 | 2 | 15.9 | 5.76 |

**Additional Figure S1.**

2D-Gel electropherograms showing numbered protein spots identified by LC-MS/MS in

1. Crude plasma. (Protein identifiers are presented in Table S1)
2. Depleted plasma. (Protein identifiers are presented in Table S1)
3. Close-up images of (CLI) Spot ID54 in two representative DIGE gels for each group.

CC = Community Controls; UM = Uncomplicated Malaria; CM = Cerebral Malaria; SMA = Severe Malaria Anaemia.


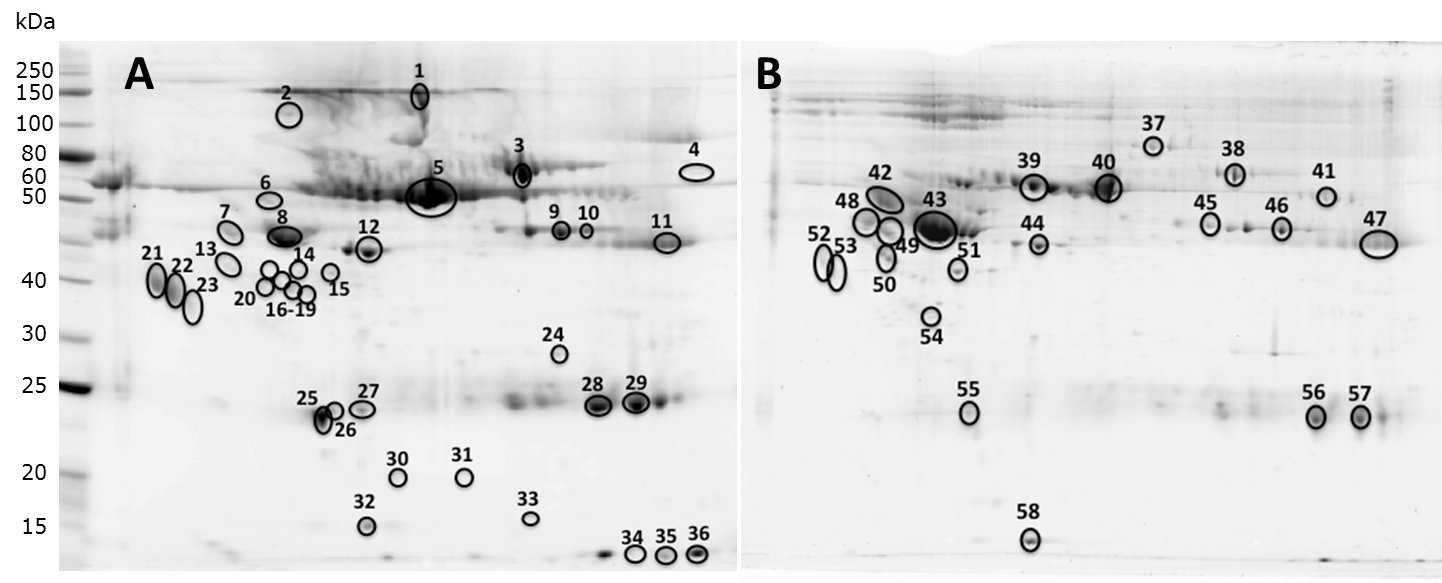


pI 3

10

pI 3

10


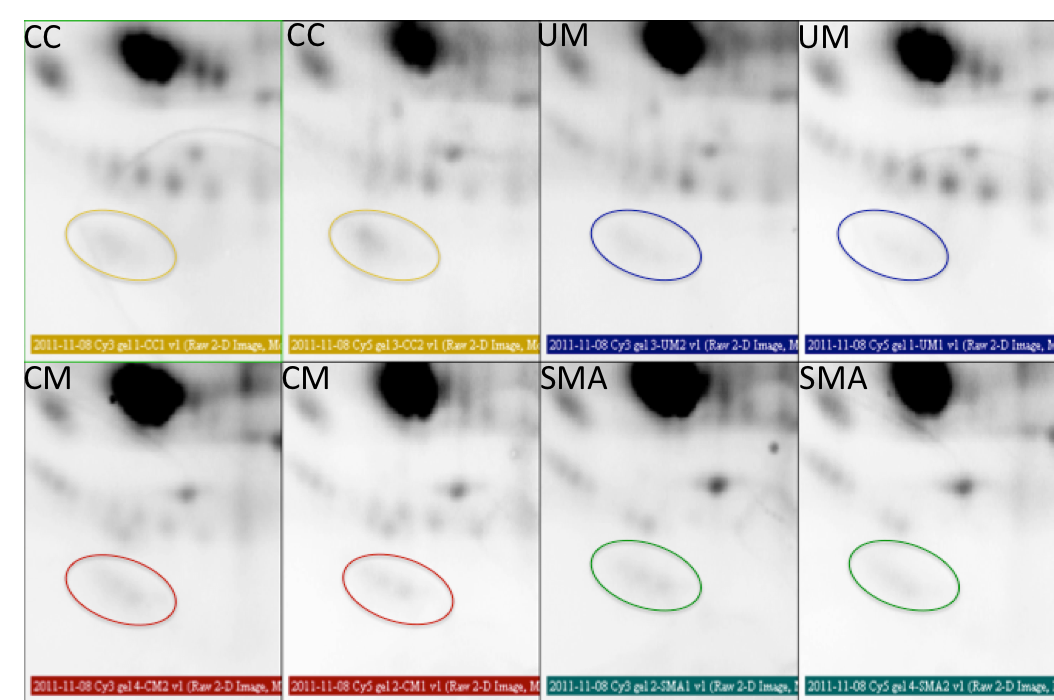


**C**
